# A Comparison of Two Hybrid Closed-Loop Systems in Italian Children and Adults With Type 1 Diabetes

**DOI:** 10.3389/fendo.2021.802419

**Published:** 2022-01-18

**Authors:** Marta Bassi, Marsida Teliti, Marilea Lezzi, Arianna Iosca, Marina Francesca Strati, Luca Carmisciano, Giuseppe d’Annunzio, Nicola Minuto, Davide Maggi

**Affiliations:** ^1^ Department of Neuroscience, Rehabilitation, Ophthalmology, Genetics, Maternal and Child Health, University of Genoa, Genoa, Italy; ^2^ Department of Pediatrics, Istituto di Ricovero e Cura a Carattere Scientifico (IRCCS) Istituto Giannina Gaslini, Genoa, Italy; ^3^ Department of Internal Medicine and Medical Specialties (DiMI), University of Genoa, Genoa, Italy; ^4^ Department of Helath Science (DiSSAL), University of Genoa, Genoa, Italy; ^5^ Diabetes Clinic, Istituto di Ricovero e Cura a Carattere Scientifico (IRCCS) Ospedale Policlinico San Martino Genoa, Genoa, Italy

**Keywords:** AHCL (advanced hybrid closed loop), type 1 diabetes, CGM (continuous glucose monitoring), CSII (continuous subcutaneous insulin infusion), TIR (time in range)

## Abstract

Tandem Control-IQ and Minimed 780G represent the most Advanced Hybrid Closed Loop (AHCL) systems currently available in pediatric and adult subjects with Type 1 Diabetes (T1D). We retrospectively compared clinical and continuous glucose monitoring data from 51 patients who upgraded to Minimed 780G system and have completed 1-month observation period with data from 39 patients who upgraded to Tandem Control-IQ. Inverse probability weighting was used to minimize the basal characteristics imbalances. Both AHCL systems showed a significant improvement in glycemic parameters. Minimed 780G group achieved higher TIR increase (p= 0.004) and greater reduction of blood glucose average (p= 0.001). Tandem Control-IQ system significantly reduced the occurrence of TBR (p= 0.010) and the Coefficient of Variation of glucose levels (p= 0.005). The use of ACHL systems led to a significant improvement of glycemic control substantially reaching the International recommended glycemic targets. Minimed 780G appears to be more effective in managing hyperglycemia, while Tandem Control-IQ seems to be more effective in reducing time in hypoglycemia.

## Introduction

The management of type 1 diabetes (T1D) has changed substantially over the past five years. Evolving technologies offer the potential to improve glycemic control by reducing burden and risk of hypoglycemia and hyperglycemia and decrease the rate of diabetes complications (1–3). Since FDA approved the first Hybrid Closed Loop (HCL) system in September 2016, further advanced devices have been commercialized. These systems integrate insulin infusion with continuous glucose monitoring (CGM) (4, 5).

Advanced Hybrid Closed Loop (AHCL) systems combine automated basal rate and correction boluses to keep glycemic values in a target range (6, 7). Patients are only re-quired to estimate carbohydrate consumption for meal boluses.

In Italy two AHCL systems are provided by the national health system and approved for both pediatric and adult patients: the Tandem t:slim X2 Control IQ™ system (Tandem Inc., San Diego, California); and the Minimed™ 780G system (Minimed Medtronic, Northridge, California).

The Minimed 780G pump is integrated with the Guardian Sensor 3 (Medtronic, Northridge, California), the Tandem Control-IQ is associated with the Dexcom G6 (Dexcom Inc., San Diego, CA) system.

These two systems use different glycemic targets: 100, 110 or 120 mg/dl for Minimed 780G system (personalization based on patients’ choice); 112.5-160 mg/dl for Tandem Control-IQ. The Minimed 780G system has an “exercise” target at 150 mg/dl, similar to Tandem (140-160 mg/dl); Control-IQ has a fixed “sleep” target mode of 112.5-120 mg/dl. In the sleep mode the system does not deliver correction boluses.

The systems adopt different algorithms for correction boluses. In particular, the Minimed 780G system can carry out up to 12 correction boluses per hour and decide the basal rate automatically. The Tandem system is able to deliver a maximum of one correction bolus per hour and modifies the basal profile based on a 30-minute prediction horizon of glucose levels.

Furthermore, the Minimed 780G system calculates by itself the daily insulin total in order to define the insulin sensitivity factor. The patient can only change the insu-lin-to-carbohydrate (I/CHO) ratios for meal boluses and the active insulin time.

The Tandem Control IQ system uses the patient’s weight and daily insulin total to cal-culate the basal insulin rate. The user can change the basal rate, I:C ratios for meal boluses and insulin sensitivity factor.

Currently, the parameters indicating a good glycemic control are evaluated through the analysis of CGM data (8). A good glycemic control is defined by the International Consensus as: Time in Range (TIR) (70-180 mg/dl) > 70%, Time Below Range (TBR) (<70 mg/dl) < 4%, TBR<54 mg/dl < 1%, Time Above Range (TAR) (>180 mg/dl) < 25%, TAR>250 mg/dl <1% (9).

Data from early studies about Tandem Control-IQ or Minimed 780G in adolescents and adults with type 1 diabetes are encouraging in terms of glycemic outcomes and patient satisfaction (10, 11). The results of a one-year real-world use of Tandem Control-IQ system (12) confirmed the conclusions reached by the two pivotal trials (10, 11). The use of Control-IQ technology increased time in range (TIR 70–180 mg/dl) from 63.2% at baseline to 73.5% at 12 months (p < 0,001) in a sample of 7813 patients with T1D (12).

Two multicenter randomized trials in children, adolescents and adults demonstrated the efficacy of Control-IQ compared to sensor-augmented pump (control group) (13, 14).

A recent study in children, that participated in a virtual educational camp, demonstrates an improvement of TIR with Control-IQ technology in comparison with Basal-IQ, a predictive low-glucose suspend (PLGS) algorithm (15). Likewise, the use of Minimed 780G system led to a reduction of time above range (TAR > 180 mg/dl) without increasing time below range (TBR < 70 mg/dl) in 52 patients (aged 15-65 years), that were well-controlled and experienced Minimed 640 users (16). These findings are supported by other evidence that demonstrates safety and effectiveness in controlling day and night glucose levels (17–19). The real-world use of Minimed 780G also provides an increased level of patient satisfaction (20).

Despite the emerging evidence on the efficacy of ACHL systems, there are no clinical studies comparing data on benefits and glycemic outcomes of Minimed 780G and Tandem Control-IQ.

The aim of this study was to compare glycemic control between Minimed 780G and Tandem Control-IQ users one month after starting the therapy.

## Materials and Methods

A retrospective dual center study was performed from October 2020 to April 2021. A total of 90 T1D patients, followed at the IRCCS G.Gaslini Pediatric Diabetology Center (Genoa, Italy) or San Martino Polyclinic Hospital Diabetes Clinic (Genoa, Italy), were upgraded to Minimed 780G or Tandem Control-IQ. The two diabetes centers involved in the study belong to the same university. and follow the same guidelines in terms of patient management and therapeutic education.

Patients were enrolled according to the following inclusion criteria: T1D diagnosis at least one-year prior to the study, insulin therapy with CSII or MDI, use of CGM with at least one-months’ worth of data before and after starting the AHCL. Patients who dropped out of the AHCL system before one month of use were excluded.

The observational period has been divided in Time 0 (T0 – start day of AHCL) and Time 1 (T1 – one month of ACHL therapy). At T0, the following data were collected for each patient: demographical data (sex, date of birth, age), age at clinical onset of T1D, duration of disease and previous type of insulin therapy. At T0 and T1 we compared: glycated hemoglobin (HbA1c) values, and blood glucose control data of the previous 14 days, through the CGM data download (each patient participating in the study wore CGM in the 14 days before T0). The following parameters were evaluated: TIR, TAR, TAR > 250 mg/dl, TBR, TBR < 54 mg/dl, Coefficient of Variation (CV), Standard Deviation (SD) and percentage of sensor use. In this study, the analyses at T1 were performed with both systems in Auto Mode and by excluding the first two-weeks of the run-in phase. CGM data were collected using data download platforms based on the technology used (Carelink™, Tidepool™, Dexcom Clarity™).

All patients (or parents if age < 18 years) provided a written informed consent in accordance with EU regulation 2016/679 to participate in the study.

Mean and SD were used to summarize continuous variables, whereas count and percentages were used for categorical variables. A separate linear regression model with baseline offset was used to estimate treatment effects on TIR, TAR, TAR>250, TBR, TBR<54, average glucose levels, SD and CV. Inverse probability weighting (IPW) was used to adjust estimates for potential baseline confounders: the subjects are weighted by the inverse of their probability to be assigned to their treatment (21). IPW was estimated by fitting a logistic regression model with the most unbalanced patients’ characteristics between the two-treatment groups (TIR, HbA1c and age). For our primary analysis we assumed there was no interaction between current and previous treatment, we then ran an exploratory analysis to test this assumption. The IPW was calculated in the following way: I) a logistic regression model was fitted to determine the propensity of subjects to be treated with their treatment (either Minimed 780G or Tandem Control-IQ); II) based on the estimated model, probabilities were calculated for each participant; and III) the inverse of the probabilities was applied as weights in the linear regression models. IPW adjusted estimates were reported. In head-to-head comparisons, when Minimed 780G or Tandem Control-IQ subgroup-specific p values were reported we applied the Bonferroni adjustment for multiple comparisons.

To test the treatment effect difference among age groups, an interaction term between the treatment group and the age group was included in each regression model.

As sensitivity analysis, patients in treatment groups (Minimed 780G or Tandem Control-IQ) were matched to minimize the imbalance of the baseline characteristics. We used a 1:1 propensity score match performed with nearest neighbour algorithm on the most unbalanced characteristics of the patients between the two-treatment groups (TIR, HbA1c and age). Subsequent analyses were performed with and without adjustments for baseline characteristics that remained unbalanced after the matching (namely HbA1c) to allow further adjustments for residual confounding (**Supplementary Tables 1** **–** **3**).

An interaction term between current and previous treatment was considered to investigate the presence of any subgroup-specific effects and the p for interaction was reported for exploratory purposes. Two-sided α less than 0.05 was considered statistically significant. All statistical analyses were performed using R software version 4.0.2 (2020-06-22).

## Results

We collected the data of 90 patients (aged 5-65 years) from two Regional Pediatric and Adult Diabetology Centers (IRCCS G.Gaslini and San Martino Polyclinic Hospital, Genoa, Liguria). 51 of these patients (23 children and adolescents < 18 years) carried the Minimed 780G system and 39 (24 children and adolescents < 18 years) the Tandem-Control IQ system. The clinical characteristics of the population at baseline (T0) are summarized in **Table 1**.

**Table 1:** Patient characteristics at baseline (T0), overall and by treatment group.

	Overall	Minimed 780G	Control-IQ	p
	N = 90	N = 51	N = 39	
Male, N (%)	47 (52.2)	29 (56.9)	18 (46.2)	0.427
Age, Mean (SD)	20.7 (13.2)	24.4 (15.7)	16.0 (6.5)	0.002
5-11 years, N (%)	26 (28.9)	14 (27.5)	12 (30.8)	0.227
12-18 years, N (%)	21 (23.3)	9 (17.6)	12 (30.8)	
> 18 years, N (%)	43 (47.8)	28 (54.9)	15 (38.4)	
Age at disease onset, Mean (SD)	9.7 (7.8)	11.2 (9.4)	7.8 (4.3)	0.041
Disease duration (yrs), Mean (SD)	11.0 (9.4)	13.2 (10.3)	8.2 (7.1)	0.010
HbA1c (%), Mean (SD)	7.5 (0.9)	7.8 (1.0)	7.1 (0.7)	0.002
TIR (%), Mean (SD)	55.7 (15.5)	52.4 (16.2)	59.6 (13.9)	0.031
TAR (%), Mean (SD)	25.3 (10.4)	25.1 (11.4)	25.5 (9.2)	0.856
TAR250mgdl (%), Mean (SD)	14.7 (12.9)	16.8 (15.0)	12.3 (9.4)	0.107
TBR (%), Mean (SD)	2.1 (1.9)	2.0 (1.7)	2.2 (2.1)	0.587
TBR54mgdl (%), Mean (SD)	0.6 (1.0)	0.6 (1.0)	0.7 (1.0)	0.558
Average glucose (mg/dl) (SD)	174.9 (32.2)	181.5 (36.1)	167.2 (25.1)	0.040
SD (mg/dl), Mean (SD)	63.5 (15.8)	64.7 (17.7)	61.1 (10.8)	0.390
CV (%), Mean (SD)	36.2 (6.1)	35.8 (5.8)	36.8 (6.5)	0.462
Time Active CMG (%), Mean (SD)	88.4 (17.7)	86.5 (17.2)	90.6 (18.3)	0.291
Previous treatment, N (%)				<0.001
MDI	17 (18.9)	10 (19.6)	7 (18.0)	
SAP	22 (24.4)	14 (27.5)	8 (20.5)	
PLGS	34 (37.8)	10 (19.6)	24 (61.5)	
HCL	17 (18.9)	17 (33.3)	0 (0.0)	

At baseline, patients upgraded to Minimed 780G versus patients upgraded to Tandem Control-IQ presented unbalanced characteristics. Tandem users were younger (mean age 16.0 years vs 24.4; p=0.002), with earlier disease onset (mean age 7.8 years vs 11.2; p = 0.041) and shorter disease duration (mean 8.2 years vs 13.2; p = 0.041). Patients in Tandem group compared to patients in Minimed 780G group had lower baseline HbA1c (7.1% vs 7.8%; p=0.002); higher TIR (59.6% vs 52.4%; p=0.031) and lower average glucose (167.2mg/dl vs 181.5mg/dl; p=0.040).

The whole study population has been previously treated with MDI (18.0%), Sensor Augmented Pumps (SAP) (24.7%), PLGS (38.2%) or HCL pumps (19.1%).

Overall, patients reported a significant improvement from T0 to T1 in TIR (+14.6%, p<0.001), TAR (-5.7%, p<0.001), TAR > 250 mg/dl (-7.7%, p<0.001), average glucose value (-19.5 mg/dl, p<0.001) and SD (-12.9 mg/dl, p<0.001); while no significant differences were observed in TBR 54-70 mg/dl and severe hypoglycemia <54 mg/dl (**Table 2**).

**Table 2:** Summary of overall treatment effect.

	T1 – T0	p
	Mean difference (95%CI)	
TIR (%)	14.6 (11.4, 17.9)	<0.001
TAR (%)	-5.7 (-7.8, -3.5)	<0.001
TAR250mgdl (%)	-7.7 (-10.3, -5.1)	<0.001
TBR (%)	-0.2 (-0.6, 0.2)	0.429
ITBR54mgdl (%)	-0.2 (-0.4, 0.0)	0.076
Average Glucose (mg/dl)	-19.5 (-26.6, -12.4)	<0.001
SD (mg/dl)	-12.9 (-16.9, -9.0)	<0.001
CV (%)	-3.0 (-4.9, -1.0)	0.003
%Time Active CGM	2.0 (-1.7, 5.6)	0.281

Despite both AHCL systems led to an improvement in glycemic control at T1, we observed a significant difference in the treatment effects in Minimed 780G group compared to Tandem Control-IQ system (**Table 3**).

**Table 3:** Treatment effects by group.

Parameter	Group	Treatment effect	Control-IQ vs Minimed 780G	p
		Mean difference (95%CI)		
TIR (%)	Minimed 780G	19.1 (14.3, 23.9)	-9.3 (-15.5, -3.1)	0.004*
	Control-IQ	9.8 (5.9, 13.7)		
TAR (%)	Minimed 780G	-7.3 (-10.6, -4.1)	3.5 (-0.8, 7.8)	0.109
	Control-IQ	-3.8 (-6.7, -1.0)		
TAR 250mgdl (%)	Minimed 780G	-9.9 (-13.9, -5.9)	4.6 (-0.5, 9.8)	0.079
	Control-IQ	-5.3 (-8.5, -2.1)		
TBR (%)	Minimed 780G	0.37 (-0.21, 0.94)	-1.0 (-1.8, -0.3)	0.010*
	Control-IQ	-0.68 (-1.23, -0.12)		
TBR 54mgdl (%)	Minimed 780G	-0.08 (-0.28, 0.12)	-0.2 (-0.6, 0.2)	0.316
	Control-IQ	-0.27 (-0.63, 0.09)		
Average glucose (mg/dl)	Minimed 780G	-31.0 (-41.3, -20.6)	23.9 (10.7, 37.0)	0.001*
	Control-IQ	-7.1 (-14.9, 0.7)		
SD (mg/dl)	Minimed 780G	-11.4 (-16.3, -6.5)	-4.6 (-12.9, 3.8)	0.276
	Control-IQ	-16.0 (-23.2, -8.7)		
CV (%)	Minimed 780G	-0.32 (-1.98, 1.35)	-5.4 (-9.1, -1.7)	0.005*
	Control-IQ	-5.68 (-9.33, -2.03)		
%Time Active CGM	Minimed 780G	2.23 (-1.74, 6.19)	-0.5 (-7.8, 6.8)	0.891
	Control-IQ	1.72 (-5.03, 8.47)		

*Treatment effect, Control-IQ vs Minimed 780Gand p columns are adjusted for baseline confounders with the inverse probability weighting strategy.

The IPW adjusted estimates showed for the Minimed 780G group a higher TIR increase (respectively +19.1% vs +9.8%; p = 0.004) and a greater reduction of blood glucose average (respectively -31 mg/dl vs -7.1mg/dl; p = 0.001), while Tandem Control-IQ achieved less time spent in TBR (respectively -0.68% vs +0.37%; p = 0.010) and greater CV reduction (respectively -5.68% vs -0.32%; p = 0.005).

No significant differences were observed between the treatment effect of Minimed 780G and the treatment effect of Tandem Control-IQ on TAR, TAR>250mg/dl, TBR<54mg/dl, SD and the proportion of active CGM time.

The analysis on the efficacy of the two AHCL systems in terms of CGM metrics did not show significant evidence of heterogeneity of the results between the age subgroups. The variables significantly associated with a difference in efficacy of the two treatments in the main analysis (TIR, TBR, average glucose and CV) are consistent in the direction of the estimates in all subgroups (**Supplemetary Table 4**).

Exploratory subgroup analysis suggests a limited impact on glycemic parameters determined by the previous therapy (**Supplementary Table 5**).

## Discussion

The aim of this study was to compare real-life glycemic control data between Minimed 780G and Tandem Control-IQ users one month after starting the system. To the best of our knowledge, in clinical setting this is the first study to compare efficacy and safety of the AHCL systems currently available in Italy in children and adults with T1D.

Recent real-world studies have only examined the performance of each ACHL system. As shown by Messer et al. in 191 children and young adults (median age 14 years), Control-IQ system improved TIR from 57% to 66% after 6-months; time spent in hypoglycemia (< 70 mg/dl) decreased from baseline to 6-months; time spent in severe hypoglycemia (<54 mg/dl) did not change (22). Breton et al. in a large data set study confirmed the TIR improvement in 7813 TD1 subjects (62% vs 72%). In parallel, time < 70 mg/dl remained low at – 1% throughout the year (12).

Meanwhile Beato-Vibora et al. reported an immediate improvement in TIR 70-180 mg/dl from 67.3% to 79.6% in the first 30 days after the initiation of the Minimed 780G ACHL system in adults and adolescents with T1D (16). No difference in time in hypoglycemia < 70 or 54 mg/dl were seen at 2 weeks or 1 month. The real-world benefits of the Medtronic 780G system in terms of glycemic control were maintained after 3 months of use of the system (20). These data agree with previous trials of the Minimed 780 G system (17, 19). In their multicenter, randomized crossover study Bergenstal et al. found a 4% increase in TIR 70-180 mg/dl compared to Minimed 670G users after 3 months (17). In the pivotal study, Collyns et al. found a 12.5% improvement in TIR 70-180 mg/dl after 1 month of use in children and adults (19). A recently published study by Da Silva et al. showed the real-life report of 4120 Minimed 780G users and showed the achievement of glycemic treatment goals: GMI <7.0% and TIR > 70% in most patients (23).

In our study we compared Minimed 780G and Tandem Control-IQ systems. Both systems showed a significant improvement in glycemic parameters after a month of therapy (T1) and substantially reached the targets recommended by International Consensus on Time in Range (TIR > 70%, TBR<70 mg/dl < 4%, TBR<54 mg/dl<1%, TAR>180 mg/dl <25%, TAR>250 mg/dl <5%) (9).

However, significant differences in the treatment effects were observed. Tandem Control-IQ system significantly reduced time spent in TBR 70-54 mg/dl (-0.68% vs +0.37% p=0.010) and CV (- 5.68% vs – 0.32% p 0.005), whereas Minimed 780G improved TIR (+19.1% vs +9.8% P = 0.004) and blood glucose average (-31% vs -7.1% P= 0.001). No significant differences were observed in the other CGM parameters. In both cases adherence to the sensor use was adequate (> 85%) (9, 24, 25).

As an additional exploratory analysis, we compared the glycemic control of patients in relation to the type of therapy previously used to assess if it impacts the efficacy of these two systems. The subgroups of previous therapy (MDI, SAP, PLGS, HCL) had heterogeneous patient characteristics and a small number of patients, which may have resulted in a statistically underpowered analysis (**Supplementary Table 4**). Aware of the aforementioned limits, we observed no significant impact on glycemic parameters determined by the previous therapy.

Given the absence of other comparative studies in the real-world settings, we can speculate that the Minimed 780G system is more effective in managing hyperglycemia. This result could be obtained by customization of the glycemic target and active insulin time and the possibility to deliver corrective boluses more frequently. This leads to better results in terms of TIR but causes a slight increase of time in hypoglycemia. Glycemic variability is known to be correlated with the risk of hypoglycemia. CV threshold of 36% is used to define stable and unstable glycemia in diabetes because, beyond this limit, the frequency of hypoglycemia is significantly increased (26, 27). Therefore, in the Control-IQ group, the improvement in CV leads to a lower TBR and likely to more stable blood glucose values. The significant reduction of TBR is very important from a clinical point of view. Clinicians place the prevention of hypoglycemia among the primary objectives of therapeutic management, due to the fear of this event itself, due to the inevitable consequences it implies on therapeutic choices, but also for the possible long-term consequences caused by prolonged periods of hypoglycemia.

One possible limitation of our study may be represented by the short period of follow-up, but as shown by Breton et al., regarding Tandem Control-IQ (12) and by Petrovski et al., regarding Minimed 670G (28), we can assume that TIR improvement observed during the first two weeks of analysis will be maintained throughout the following year. It is nevertheless true that, the retrospective observational nature of the study limits interpretation and generalizability of our results.

Strengths and at the same time possible limitations of this study are the broad age-range of the sample, going from school-aged children to adults, and heterogeneity of previous therapeutic schemes. The real-life clinical practice setting is an important strength of our study.

In conclusion this is the first study to compare the Minimed 780G with Tandem Control-IQ systems. In summary, data of this first study showed that the Minimed 780G system seems more effective in managing hyperglycemia, while Tandem Control-IQ reduces the number of hypoglycemic episodes and glucose variability. Aside from these little differences between the two systems, it is clear that they both substantially reach the glycemic target and that further studies with a larger population and a longer follow-up period are needed to draw conclusions about the differences between the two systems. Understanding the strength and limitations of AHCL devices could be useful for “proper candidate selection” and tailoring insulin pump therapy.

## Data Availability Statement

The original contributions presented in the study are included in the article/**Supplementary Material**. Further inquiries can be directed to the corresponding author.

## Author Contributions

MB designed the study and wrote the manuscript. MT designed the study and wrote the manuscript. ML researched data. AI researched data. MS researched data and reviewed the manuscript. LC did statistical analysis. GD’A reviewed the manuscript and contributed to the discussion. NM designed the study and contributed to the discussion. DM designed the study and contributed to the discussion. All authors contributed to the article and approved the submitted version.

## Conflict of Interest

The authors declare that the research was conducted in the absence of any commercial or financial relationships that could be construed as a potential conflict of interest.

The reviewer RS declared a past collaboration with several of the authors GD and NM to the handling editor.

## Publisher’s Note

All claims expressed in this article are solely those of the authors and do not necessarily represent those of their affiliated organizations, or those of the publisher, the editors and the reviewers. Any product that may be evaluated in this article, or claim that may be made by its manufacturer, is not guaranteed or endorsed by the publisher.
