# Supplementary material for: A Comparison of Two Hybrid Closed-Loop Systems in Italian Children and Adults With Type 1 Diabetes
Source: Front Endocrinol (Lausanne). 2022 Jan 18;12:802419. doi: 10.3389/fendo.2021.802419 (PMC8805205; doi:10.3389/fendo.2021.802419)
Supplement: Supplementary file 1 [file Table_1.docx]

## Propensity score match

As sensitivity analysis, patients in treatment groups (Minimed 780G or Control-IQ) were matched to minimize the imbalance of the baseline characteristics (Supplementary Table 1). We used a 1:1 propensity score match performed with nearest neighbor algorithm on the most unbalanced characteristics of the patients between the two-treatment group (TIR, HbA1c and age). Subsequent analyses were performed with and without adjustments for baseline characteristics that remained unbalanced after the matching (namely HbA1c) to allow further adjustments for residual confounding. For the purpose of matching, we assumed the treatment effect to be independent from previous T1D treatments, to explore this assumption a separate sensitivity analysis was produced.

The sensitivity analysis using propensity score matching estimated treatment effect differences between Minimed 780G and Control-IQ close to the ones estimated by the primary IPW analysis but confirming only TBR difference as significant (Supplementary Table 3).

One of the major limits of this method is the loss in statistical power due to patient exclusion, which may result in inflated p values.

### Supplementary Table 1. Characteristics of matched population at T0.

|  | **MINIMED 780G,**  **N = 32** | **TANDEM,**  **N = 32** | p |
| --- | --- | --- | --- |
| Male, N (%) | 17 (53.1) | 16 (50.0) | 0.999 |
| Age, Mean (SD) | 19.47 (10.94) | 15.81 (6.65) | 0.111 |
| 5-11 years, N (%) | 12 (37.5) | 11 (34.4) | 0.468 |
| 12-18 years, N (%) | 5 (15.6) | 9 (28.1) |  |
| > 18 years, N (%) | 15 (46.9) | 12 (37.5) |  |
| Age at disease onset, Mean (SD) | 8.90 (6.05) | 7.38 (4.34) | 0.251 |
| Disease duration, Mean (SD) | 10.57 (7.84) | 8.43 (7.32) | 0.264 |
| HbA1c (%), Mean (SD) | 7.52 (0.68) | 7.14 (0.71) | 0.031 |
| TIR (%), Mean (SD) | 54.09 (16.35) | 58.22 (14.17) | 0.285 |
| TAR (%), Mean (SD) | 25.74 (10.42) | 25.34 (9.46) | 0.872 |
| TAR250mgdl (%), Mean (SD) | 15.85 (15.75) | 13.72 (9.55) | 0.516 |
| TBR (%), Mean (SD) | 1.97 (1.63) | 2.25 (2.24) | 0.581 |
| TBR54mgdl (%), Mean (SD) | 0.51 (0.64) | 0.72 (1.07) | 0.348 |
| Average glucose (mg/dl) (mean (SD)) | 178.26 (40.23) | 169.19 (26.10) | 0.291 |
| SD (mg/dl), Mean (SD) | 63.84 (17.91) | 63.06 (10.85) | 0.867 |
| CV (%), Mean (SD) | 35.65 (5.26) | 37.66 (6.59) | 0.185 |
| %Time active CGM (mean (SD)) | 89.30 (10.97) | 91.63 (19.32) | 0.560 |
| Insulin delivery o Previous treatment, N (%) |  |  | <0.001 |
| MDI | 4 (12.5) | 6 (18.8) |  |
| SAP | 12 (37.5) | 7 (21.9) |  |
| PLGS | 7 (21.9) | 19 (59.4) |  |
| HCL | 9 (28.1) | 0 (0.0) |  |
