# Supplementary material for: A Comparison of Two Hybrid Closed-Loop Systems in Italian Children and Adults With Type 1 Diabetes
Source: Front Endocrinol (Lausanne). 2022 Jan 18;12:802419. doi: 10.3389/fendo.2021.802419 (PMC8805205; doi:10.3389/fendo.2021.802419)
Supplement: Supplementary file 2 [file Table_2.docx]

**Supplementary Table 2. Crude treatment effects by treatment group**

**(matched population, N = 64)**

| Parameter | Group | Treatment effect  Mean difference (95%CI) | Control-IQ vs  Minimed 780G | p |
| --- | --- | --- | --- | --- |
| TIR (%) | Minimed-780G | 20.8 (14.7, 26.9) | -10.0 (-17.4, -2.6) | 0.009* |
|  | Control-IQ | 10.8 (6.4, 15.2) |  |  |
| TAR (%) | Minimed 780G | -8.0 (-11.7, -4.3) | 2.9 (-1.7, 7.6) | 0.213 |
|  | Control-IQ | -5.0 (-8.0, -2.0) |  |  |
| TAR  250mgdl (%) | Minimed 780G | -11.6 (-17.2, -6.0) | 6.5 (0.1, 13.0) | 0.047* |
|  | Control-IQ | -5.1 (-8.5, -1.7) |  |  |
| TBR (%) | Minimed-780G | 0.44 (-0.23, 1.12) | -1.21 (-2.07, -0.36) | 0.006* |
|  | Control-IQ | -0.77 (-1.33, -0.20) |  |  |
| TBR  54mgdl (%) | Minimed 780G | -0.01 (-0.25, 0.22) | -0.27 (-0.72, 0.17) | 0.222 |
|  | Control-IQ | -0.29 (-0.68, 0.10) |  |  |
| Average  glucose (mg/dl) | Minimed 780G | -34.5 (-49.7, -19.3) | 23.5 (6.7, 40.2) | 0.007* |
|  | Control-IQ | -11.1 (-18.9, -3.3) |  |  |
| SD (mg/dl) | Minimed 780G | -13.7 (-19.9, -7.5) | 2.5 (-7.5, 12.5) | 0.618 |
|  | Control-IQ | -11.2 (-18.8, -3.6) |  |  |
| CV (%) | Minimed 780G | -0.99 (-2.89, 0.92) | -2.24 (-5.48, 1.01) | 0.173 |
|  | Control-IQ | -3.22 (-5.88, -0.57) |  |  |
| %Time  Active CGM | Minimed 780G | 1.47 (-2.64, 5.58) | -0.39 (-8.34, 7.57) | 0.923 |
|  | Control-IQ | 1.08 (-5.84, 8.00) |  |  |

** significant (p<0.05)*
