# Supplementary material for: A Comparison of Two Hybrid Closed-Loop Systems in Italian Children and Adults With Type 1 Diabetes
Source: Front Endocrinol (Lausanne). 2022 Jan 18;12:802419. doi: 10.3389/fendo.2021.802419 (PMC8805205; doi:10.3389/fendo.2021.802419)
Supplement: Supplementary file 3 [file Table_3.docx]

**Supplementary Table 3. Adjusted treatment effects by treatment group**

**(matched population, N = 64)**

| Parameter | Group | Treatment effect  Mean difference (95%CI) | Control-IQ vs  Minimed 780G | p |
| --- | --- | --- | --- | --- |
| TIR (%) | Minimed 780G | 18.1 (12.7, 23.5) | -7.0 (-14.2, 0.2) | 0.058 |
|  | Control-IQ | 11.3 (6.6, 15.9) |  |  |
| TAR (%) | Minimed 780G | -8.0 (-11.9, -4.0) | 3.1 (-1.8, 8.0) | 0.211 |
|  | Control-IQ | -4.9 (-8.0, -1.7) |  |  |
| TAR  250mgdl (%) | Minimed 780G | -8.5 (-12.6, -4.3) | 2.9 (-2.9, 8.8) | 0.317 |
|  | Control-IQ | -5.6 (-9.2, -2.1) |  |  |
| TBR (%) | Minimed 780G | 0.28 (-0.42, 0.98) | -1.09 (-1.98, -0.19) | 0.018* |
|  | Control-IQ | -0.78 (-1.37, -0.18) |  |  |
| TBR  54mgdl (%) | Minimed 780G | -0.09 (-0.32, 0.14) | -0.25 (-0.71, 0.22) | 0.293 |
|  | Control-IQ | -0.33 (-0.73, 0.08) |  |  |
| Average  glucose (mg/dl) | Minimed 780G | -26.9 (-38.8, -14.9) | 15.3 (-0.3, 30.9) | 0.054 |
|  | Control-IQ | -11.8 (-20.0, -3.7) |  |  |
| SD (mg/dl) | Minimed 780G | -11.2 (-16.8, -5.6) | -2.8 (-12.1, 6.6) | 0.555 |
|  | Control-IQ | -13.3 (-21.2, -5.4) |  |  |
| CV (%) | Minimed 780G | -1.06 (-3.06, 0.95) | -2.42 (-5.80, 0.96) | 0.157 |
|  | Control-IQ | -3.47 (-6.26, -0.69) |  |  |
| %Time  Active CGM | Minimed 780G | 2.63 (-1.40, 6.66) | -1.09 (-9.39, 7.20) | 0.793 |
|  | Control-IQ | 1.47 (-5.82, 8.75) |  |  |

*adj: after baseline HbA1c adjustments. * significant (p<0.05).*
