# Supplementary material for: A Comparison of Two Hybrid Closed-Loop Systems in Italian Children and Adults With Type 1 Diabetes
Source: Front Endocrinol (Lausanne). 2022 Jan 18;12:802419. doi: 10.3389/fendo.2021.802419 (PMC8805205; doi:10.3389/fendo.2021.802419)
Supplement: Supplementary file 4 [file Table_4.docx]

**Supplementary Table 4.** Treatment effect difference (TANDEM vs MINIMED 780G) among age subgroups.

| Parameter | OVERALL,  N = 90 | (5,11) years,  N = 26 | (12,18) years,  N = 21 | (>18) years,  N = 43 | p* |
| --- | --- | --- | --- | --- | --- |
| TIR (%) | -9.3 (-15.5, -3.1) | -2.1 (-11.7, 7.6) | -17.5 (-40.0, 4.9) | -12.9 (-20.3, -5.6) | 0.162 |
| TAR (%) | 3.5 (-0.8, 7.8) | 1.8 (-5.2, 8.8) | 9.6 (-0.4, 19.7) | 2.7 (-4.4, 9.7) | 0.448 |
| TAR  250mgdl (%) | 4.6 (-0.5, 9.8) | 1.2 (-5.3, 7.6) | 10.8 (-11.1, 32.6) | 5.5 (-0.4, 11.4) | 0.452 |
| TBR (%) | -1.0 (-1.8, -0.3) | -1.2 (-2.8, 0.3) | -2.0 (-4.3, 0.3) | -0.5 (-1.5, 0.5) | 0.380 |
| TBR  54mgdl (%) | -0.2 (-0.6, 0.2) | -0.2 (-0.8, 0.4) | -0.6 (-2.0, 0.9) | 0.0 (-0.4, 0.5) | 0.551 |
| Average  glucose (mg/dl) | 23.9 (10.7, 37.0) | 8.4 (-7.2, 23.9) | 41.3 (-17.1, 99.8) | 29.1 (14.7, 43.5) | 0.186 |
| SD (mg/dl) | -4.6 (-12.9, 3.8) | -5.7 (-23.3, 11.8) | 7.8 (-21.8, 37.4) | -8.9 (-18.6, 0.8) | 0.320 |
| CV (%) | -5.4 (-9.1, -1.7) | -3.1 (-7.3, 1.0) | -5.4 (-12.9, 2.1) | -6.7 (-13.6, 0.3) | 0.717 |
| %Time  Active CGM | -0.5 (-7.8, 6.8) | -2.1 (-6.1, 2.0) | -9.5 (-32.6, 13.6) | 2.3 (-10.3, 14.9) | 0.518 |

*p for interaction between treatment group and age group
