# Supplementary material for: A Comparison of Two Hybrid Closed-Loop Systems in Italian Children and Adults With Type 1 Diabetes
Source: Front Endocrinol (Lausanne). 2022 Jan 18;12:802419. doi: 10.3389/fendo.2021.802419 (PMC8805205; doi:10.3389/fendo.2021.802419)
Supplement: Supplementary file 5 [file Table_5.docx]

**Supplementary Table 5**. Exploratory subgroup analysis by previous treatment

| Difference (T1-T0),  Mean (SD) | Previous  treatment | Minimed 780G | Control-IQ | p for  interaction |
| --- | --- | --- | --- | --- |
| TIR (%) | MDI | 40.86 (15.42) | 21.33 (7.26) | 0.716 |
|  | SAP | 31.62 (12.24) | 12.88 (8.48) |  |
|  | PLGS | 14.60 (11.22) | 7.62 (12.29) |  |
| TAR (%) | MDI | -10.29 (11.28) | -12.12 (9.87) | 0.253 |
|  | SAP | -16.23 (7.30) | -4.50 (8.54) |  |
|  | PLGS | 5.38 (12.15) | -4.34 (7.45) |  |
| TAR250mgdl (%) | MDI | -30.57 (23.03) | -10.00 (3.16) | 0.793 |
|  | SAP | -15.85 (12.48) | -6.25 (5.31) |  |
|  | PLGS | -5.86 (4.02) | -2.67 (10.22) |  |
| TBR (%) | MDI | 0.14 (2.85) | -0.72 (2.15) | 0.916 |
|  | SAP | 0.54 (1.71) | -1.38 (1.85) |  |
|  | PLGS | 0.25 (0.96) | -0.55 (1.26) |  |
| ITBR54mgdl (%) | MDI | 0.00 (1.15) | -0.52 (1.45) | 0.202 |
|  | SAP | 0.08 (0.76) | -0.70 (1.65) |  |
|  | PLGS | -0.18 (0.23) | -0.10 (0.45) |  |
| Average glucose (mg/dl) | MDI | -79.71 (62.68) | -27.83 (5.60) | 0.807 |
|  | SAP | -48.42 (27.70) | -7.62 (14.93) |  |
|  | PLGS | -23.20 (19.53) | -7.78 (22.45) |  |
| SD (mg/dl) | MDI | -35.80 (21.68) | -18.83 (8.06) | 0.717 |
|  | SAP | -19.67 (11.99) | -11.60 (8.91) |  |
|  | PLGS | -7.10 (8.21) | -2.56 (14.27) |  |
| CV (%) | MDI | -3.19 (8.86) | -5.28 (5.16) | 0.291 |
|  | SAP | -1.62 (4.85) | -8.93 (7.64) |  |
|  | PLGS | 0.64 (3.26) | -0.06 (5.83) |  |

Small and non-significative trends may suggest a slightly better TAR control with Tandem Control-IQ for patients previously treated with PLGS (+5.38% vs -4.34%) and with Minimed 780G for patients previously treated with SAP (-16.23% vs -4.50%); a better TBR<54mgdl control with Minimed 780G for patients previously treated with PLGS (-0.18% vs -0.10%) and with Tandem Control-IQ for patients previously treated with SAP (+0.08% vs -0.70%) or MDI (0.00% vs -0.52%); and that the larger CV effect observed for Tandem Control-IQ may be driven by patients previously treated with PLGS (Minimed 780G: +0.64% vs Tandem Control-IQ : -0.06%).
